# Cleavage of Toll-Like Receptor 9 Ectodomain Is Required for *In Vivo* Responses to Single Strand DNA

**DOI:** 10.3389/fimmu.2018.01491

**Published:** 2018-06-27

**Authors:** Ryutaro Fukui, Chikako Yamamoto, Fumi Matsumoto, Masahiro Onji, Takuma Shibata, Yusuke Murakami, Atsuo Kanno, Takuto Hayashi, Natsuko Tanimura, Nobuaki Yoshida, Kensuke Miyake

**Affiliations:** ^1^Division of Innate Immunity, Department of Microbiology and Immunology, The Institute of Medical Science, The University of Tokyo, Tokyo, Japan; ^2^Institute of Molecular Biotechnology of the Austrian Academy of Sciences, Vienna, Austria; ^3^Department of Pharmacotherapy, Research Institute of Pharmaceutical Sciences, Musashino University, Tokyo, Japan; ^4^Laboratory of Developmental Genetics, The Institute of Medical Science, The University of Tokyo, Tokyo, Japan; ^5^Laboratory of Innate Immunity, Center for Experimental Medicine and Systems Biology, The Institute of Medical Science, The University of Tokyo, Tokyo, Japan

**Keywords:** toll-like receptor 9, proteolytic cleavage, Unc93 homolog B1, CpG-ODN, primary immune cells, *in vivo* response

## Abstract

Mouse toll-like receptor 9 (TLR9) is an endosomal sensor for single-stranded DNA. TLR9 is transported from the endoplasmic reticulum to endolysosomes by a multiple transmembrane protein Unc93 homolog B1, and proteolytically cleaved at its ectodomain. The structure of TLR9 and its biochemical analyses have shown that the proteolytic cleavage of TLR9 ectodomain enables TLR9-dimerization and TLR9 activation. However, the requirement of TLR9 cleavage *in vivo* has not been studied. We here show that the 13 amino acids deletion at the cleavage site made TLR9 resistant to proteolytic cleavage. The deletion mutation in the *Tlr9* gene impaired TLR9-dependent cytokine production in conventional dendritic cells from the mutant mice. Not only *in vitro, in vivo* production of inflammatory cytokines (TNF-α and IL-12p40), chemokine (CCR5/RANTES), and type I interferon (IFN-α) induced by administration of TLR9 ligand was also impaired. These results demonstrate that the TLR9 cleavage is required for TLR9 responses *in vivo*.

## Introduction

Toll-like receptors (TLRs) sense a variety of microbial products. Cell surface TLRs, including TLR4/MD-2, TLR1/TLR2, and TLR6/TLR2, recognize microbial membrane lipids, whereas TLR3, TLR7, TLR8, and TLR9 localize to intracellular organelles and respond to nucleic acids (NAs) (1–3). NA is a principal ligand for pathogen sensors. Self/pathogen discrimination by NA-sensing TLRs is error-prone and requires compartmentalization of NA sensing in endolysosomes, not on the cell surface. While self-derived NAs are rapidly degraded by nucleases, microbial NAs are resistant to degradation because it is encased in bacterial cell walls or viral particles. Pathogen-derived NAs, but not self-derived NAs, reach endolysosomes and stimulate TLRs.

To compartmentalize NA-sensing, two mechanisms are proposed. The first mechanism is based on TLR transportation. Trafficking of NA-sensing TLRs from the endoplasmic reticulum (ER) to endolysosomes is dependent on Unc93 homolog B1 (Unc93B1), a multiple transmembrane protein (4, 5). Without Unc93B1, NA-sensing TLRs fail to respond to nucleic acid, because all the NA-sensing TLRs remain in the ER (4, 5). Unc93B1 also plays a role in balancing TLR7 and TLR9 responses. A point mutation of aspartic acid at position 34 (D34A) renders TLR7 hyper-responsive and TLR9 hypo-responsive, leading to TLR7-dependent systemic lethal inflammation (6, 7).

The second mechanism depends on the proteolytic cleavage of TLRs in endolysosomes (8, 9). Previous studies suggest that NA-sensing by TLRs is silenced in the ER and activated only after the proteolytic cleavage of TLR ectodomains in endolysosomes. The ectodomain of TLR9, a sensor for single strand DNA (ssDNA), is cleaved by cathepsins and/or asparagine endopeptidase in endolysosomes (10, 11). Although previous studies indicate that the C-terminal fragment of TLR9 (TLR9C) alone responds to DNA (8, 9), the N-terminal fragment of TLR9 ectodomain (TLR9N) is still associated with TLR9C and required for TLR9 responses (12, 13). Recently, the structure of TLR9 complexed with ssDNA has been reported (14). The agonistic ssDNA interacts with leucine-rich repeat 1–10 in the N-terminal fragment of TLR9 and with LRR20-22 in the C-terminal fragment of another TLR9 to form ligand-dependent TLR9 dimer. Deletion of the cleavage site abolishes TLR9 responses (9), demonstrating the requirement for the proteolytic cleavage of the TLR9 ectodomain in TLR9 activation. Uncleaved TLR9 is able to bind to ssDNA but fails to form oligomers (14). These previous findings are, however, based on cell lines or purified TLR9. The role of TLR9 cleavage *in vivo* remains unclear.

In this manuscript, we found that the deletion of the 13 amino acids in the loop domain of TLR9 abolished proteolytic cleavage, and that the deletion impaired TLR9 responses to ssDNA in the dendritic cells (DCs) due to the lack of proteolytic cleavage of TLR9. Cytokine production by administration of TLR9 ligands was also impaired in the mutant mice. These results suggest that the proteolytic cleavage of TLR9 is required for the response to ssDNA *in vivo*.

## Materials and Methods

### Reagents and Antibodies

Staining buffer was prepared with 1 × PBS, 2% FBS, 0.04% NaHCO_3_, 2 mM EDTA, and 0.1% NaN_3_. For intracellular staining buffer, 0.1% saponin (Sigma, St. Louis, MO, USA) was added in staining buffer. CpG-A 1585 (5′-G*G*GGTCAACGTTGAG*G*G*G*G*G-3′, asterisks indicate phosphorothioated residues) and CpG-B 1668 (5′-TCCATGACGTTCCTGATGCT-3′, whole phosphorothioated) were synthesized by FASMAC (Atsugi, Japan) or Hokkaido System Science (Sapporo, Japan). Pam3CSK4 was purchased from EMC microcollections (Tübingen, Germany). Lipid A Re595 was purchased from Sigma (St. Louis, MO, USA). Loxoribine (7-allyl-7,8-dihydro8-oxo-guanosine) was purchased from Enzo Life Science (Farmingdale, NY, USA). Complete protease inhibitor and restriction enzymes were purchased from Sigma. Recombinant IL-3, IL-6, and stem cell factor were purchased from Wako Pure Chemical Industries (Osaka, Japan). Recombinant IFN-α and IFN-β were purchased from PBL Assay Science (Piscataway, NJ, USA). Recombinant IFN-γ, M-CSF, and GM-CSF were purchased from PeproTech (Rocky Hill, NJ, USA).

Anti-DYKDDDDK-APC, purified anti-CD16/32, anti-CD19-APC-Cy7, anti-CD11c-PE-Cy7, anti-CD8α-APC-Cy7, anti-Ly6G-PerCP-Cy5.5, anti-Ly6C-APC-Fire750, and streptavidin-PE were purchased from BioLegend (San Diego, CA, USA). Anti-CD3-BV421, anti-CD11b-BB515, anti-I-A/I-E-BV510, anti-CD4-BV786, anti-CD49b-BV786, anti-STAT1-APC, anti-pSTAT1-PE was purchased from BD (Franklin Lakes, NJ, USA). A rabbit anti-TLR9 TIR domain polyclonal antibody was developed in our laboratory. Anti-β-actin was purchased from Abcam (Cambridge, UK). Anti-mouse Ig-HRP and anti-Rat-HRP were purchased from Santa Cruz Biotechnology (Dallas, TX, USA). Anti-6xHis polyclonal antibodies were purchased from MBL (Nagoya, Japan). Protein A-HRP was purchased from GE Healthcare (Chicago, IL, USA).

### Cell Culture

Ba/F3 cells were cultured in Roswell Park Memorial Institute (RPMI) 1640 medium (GIBCO, Waltham, MA, USA) supplemented with IL-3, 10% FBS, penicillin–streptomycin–glutamine (PS/Gln, GIBCO), and 50 µM 2-ME (Nacalai, Kyoto, Japan). Bone marrow-derived macrophages (BM-macrophages) and conventional DCs (BM-cDCs) were prepared. In brief, to induce macrophages, BM cells were plated on at 4 × 10^6^ cells per 10 ml with 10% FBS–DMEM (GIBCO) supplemented with PS/Gln, 50 µM 2-ME, and 100 ng/ml of recombinant M-CSF (PeproTech) in 10-cm cell culture dishes for 7 days. To induce cDCs, BM cells were plated at 1 × 10^7^ cells per 10 ml with 10% FBS–RPMI 1640 supplemented with PS/Gln, 50 µM 2-ME, and 10 ng/ml of recombinant GM-CSF for 7 days. Culture suspension of BM-cDCs was split every 2–3 days.

### Mice

**Figure 1:**
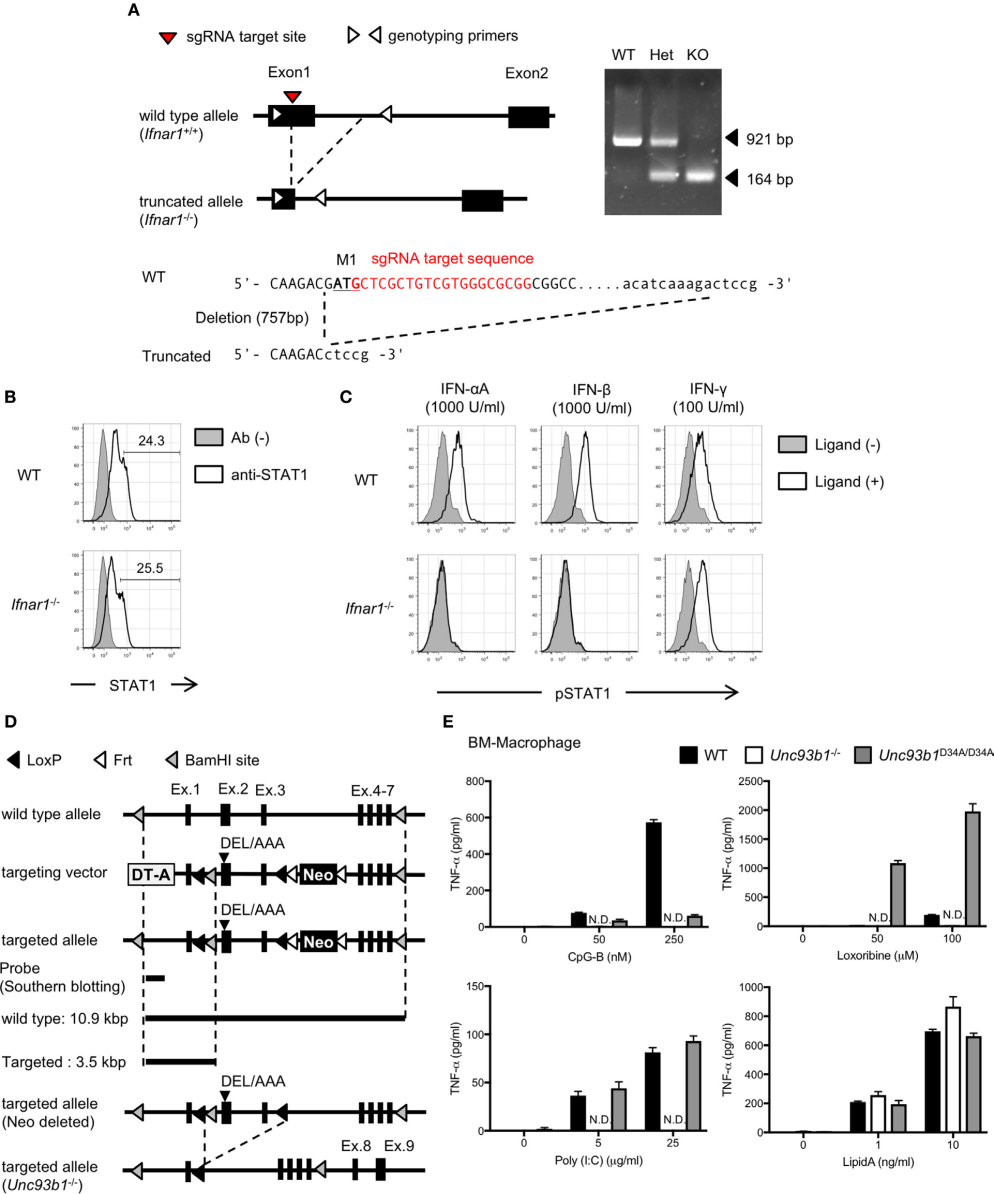
Generation of interferon alpha receptor 1 (*Ifnar1*)^−/−^ or Unc93 homolog B1 (*Unc93b1*)^−/−^ mice. **(A)** The strategy to generate *Ifnar1*^−/−^ mice and the result of genotyping. The sequences of these oligo DNA shown in the figure were listed in Table 1. **(B,C)** Intracellular staining of STAT1 or pSTAT1. Whole splenocytes were stimulated with indicated interferons and phosphorylation of STAT1 was analyzed. The cells highly express STAT1 were gated as shown in **(B)** and subjected to the analysis of phosphorylation. **(D)** The strategy to generate *Unc93b1*^−/−^ mice. Mice harboring D34A/E35A/L36A mice were generated first. Exon 2 and exon 3 of the DEL/AAA mutant mice were truncated by CAG-Cre to generate *Unc93b1*^−/−^. **(E)** TNF-α production from bone marrow-derived macrophages with toll-like receptor (TLR) ligands. The cells were stimulated with indicated concentration of TLR ligands and secreted TNF-α in culture supernatant was measured by ELISA. Cells were stimulated in triplicated wells and the mean SD was shown. At least each three mice **(B,C)** or two mice **(E)**, sex, and age matched were used. Abbreviation: ND, not detected.

**Figure 5:**
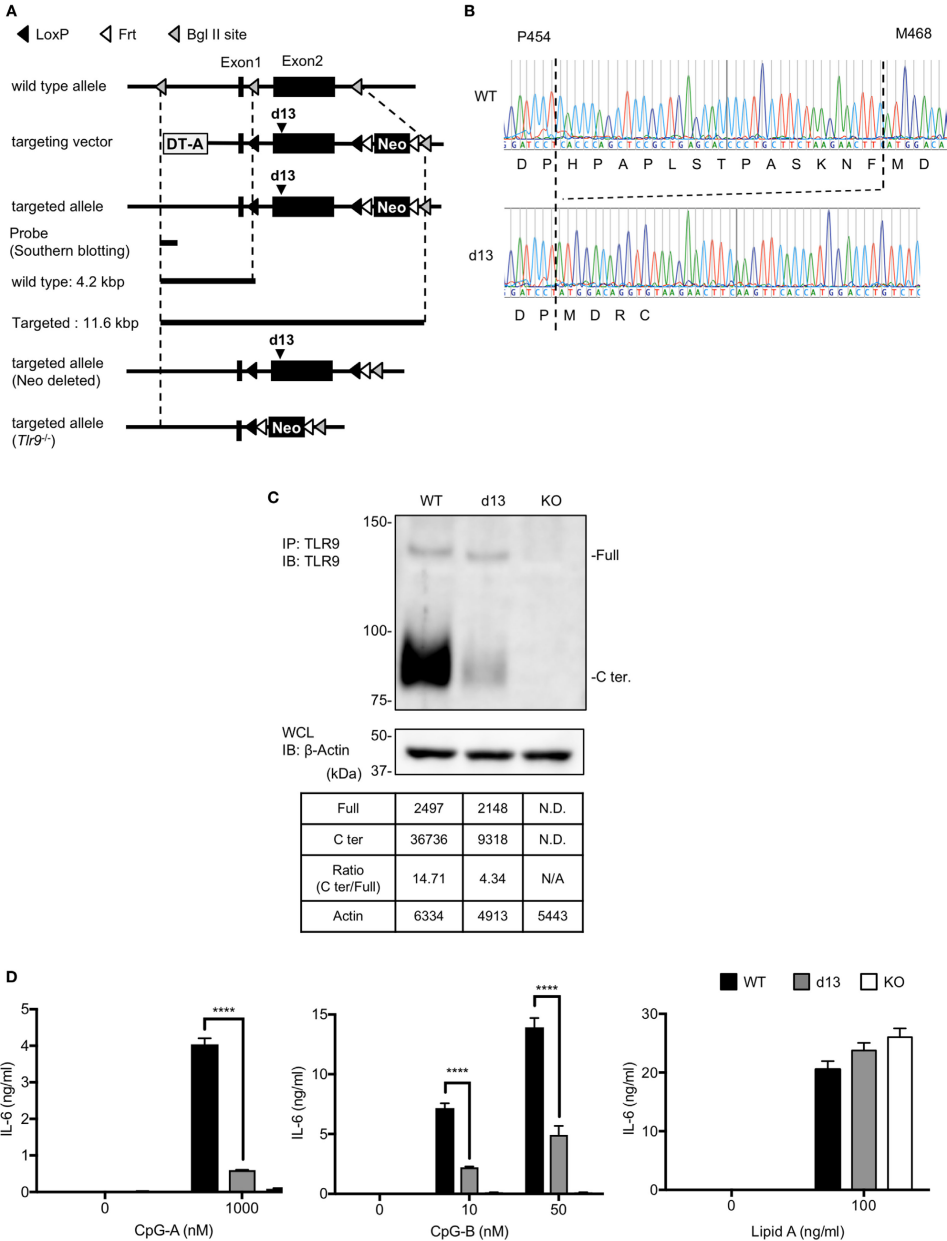
Cleavage and response of toll-like receptor 9 (TLR9) are reduced in the cells from *Tlr9*^d13/d13^ mice. **(A)** The strategy to generate *Tlr9*^d13/d13^ and *Tlr9*^−/−^ mice. **(B)** The genomic sequences of *Tlr9*^d13/d13^ mice. The sequence of DNA and the transcription are shown. **(C)** Cleavage pattern of endogenous TLR9 in bone marrow-derived conventional DCs (BM-cDCs). TLR9 in BM-cDCs obtained from WT, *Tlr9*^d13/d13^, or *Tlr9*^−/−^ mice was detected by immunoprecipitation and immunoblotting. β-actin in whole cell lysate (WCL) was detected as an internal control. The intensity of the bands was quantified and shown in the table. **(D)** BM-cDCs obtained from WT, *Tlr9*^d13/d13^, or *Tlr9*^−/−^ mice were stimulated by TLR9 or TLR4 ligand. After 24 h incubation, IL-6 in culture medium was detected by ELISA. Cells were stimulated in triplicated wells and the mean SD is shown. At least three times of experiments were performed independently **(C,D)**. Data were statistically analyzed by two-way ANOVA with multiple comparisons **(D)**. ****p < 0.0001. Abbreviations: ND, not detected; N/A, not applicable.

Wild-type C57BL/6N cr slc mice were purchased from Japan SLC (Hamamatsu, Japan). Interferon alpha receptor 1 (*Ifnar1*)^−/−^ mutant mice were generated by us with CRISPR-Cas9 system as described below (Figure 1A). *Unc93b1*^D34A/D34A^ mutant mice were generated as described previously, and backcrossed to C57BL/6N slc strain at least 21 times (6, 15). *Unc93b1*^−/−^ mice were generated by us as described below (Figure 1D). TLR9 mutant mice (*Tlr9^d13/d13^* mice, described as "d13 mice," and *Tlr9*^−/−^ mice) were generated as described below (Figure 5A). Mice used for the data were sex-matched and age-matched. These mice were kept in SPF condition. All animal experiments were approved by the Animal Research Committee of the Institute of Medical Science, The University of Tokyo, and performed in accordance with the guidelines (permission number from the committee: A17-82, A17-83, and A17-84).

### Generation of *Ifnar1*^−/−^ Mice

Single guide RNA (sgRNA) targeting *Ifnar1* was designed as shown in Figure 1A; Table 1A, and cloned into PX458 vector (addgene). By using the vector as template, complex of sgRNA and tracrRNA was synthesized by MEGAshortscript T7 Transcription Kit (Thermo Scientific, Waltham, MA, USA). mRNA for hCas9 was synthesized by mMESSAGE mMACHINE T7 ULTRA Transcription Kit (Thermo Scientific), and these RNA products were purified by MEGAclear Transcription Clean-Up Kit (Thermo Scientific). 50 ng/µl of sgRNA/tracrRNA and 100 ng/µl of mRNA for hCas9 were mixed and subjected to the injection into fertilized eggs. N0 mice were crossed with wild-type C57BL/6N mice at least five times and intercrossed to obtain homogenous mutant mice. The mutation is detected by PCR and sequencing (Figure 1A; Table 1A). The sequence of primers was shown in Table 1A.

**Table 1:** Sequences of oligo DNA for generating and genotyping of mutant mice.

(A) Sequences of oligo DNA for interferon alpha receptor 1 (*Infar1*)^**−**/**−**^ mice		
Name of oligo DNA	Sequence (5′→3′)	Application
Infar1-single guide RNA (sgRNA)1	GCTCGCTGTCGTGGGCGCGG	Cloning
Ifnar1-T7-gRNA-1-Fw	TAATACGACTCACTATAGGGGCTCGCTGTCGTGGGCGCGG	IVT
gRNA-tracr-Rv	AAAAAGCACCGACTCGGTGCCAC	IVT
T7-Cas9-Fw	TAATACGACTCACTATAGGGGACAAGAAGTACAGCATCGGCCTGGAC	IVT (hCas9)
Cas9-Rv	TCACTTTTTCTTTTTTGCCTGGCCGGCCTTTTTCGTG	IVT (hCas9)
Ifnar1-exon1-Fw1	TAGCTGCCCAGAGGTAGTCTCCAGCTC	Genotyping
Ifnar1-intron1-2-Rv2	TCTGAGTTGTCAGTTTCTCAGTGCTGTC	Genotyping

**(B) Sequences of oligo DNA for Unc93 homolog B1 (***Unc93b1***)^−/−^ mice**		

Unc93b1-loxpFw	TGAGCAGGGCAGGGAAACACAATGGGAC	Genotyping
Unc93b1-typing-Rv2	TGTGTCACAGCACTGGGGAACTTAAGTC	Genotyping

**(C) Sequences of oligo DNA for Toll-like receptor 9 (***Tlr9***)^−/−^ mice**		

Tlr9-extra	GCAATGGAAAGGACTGTCCACTTTGTG	Genotyping
Tlr9-WT	GAAGGTTCTGGGCTCAATGGTCATGTG	Genotyping
Tlr9-KO-Rv1	ATATCTGAACAGAGTGACTCAGCACGTCCTC	Genotyping

**(D) Sequences of oligo DNA for ***Tlr9***^d13/d13^ mice**		

153.NeoDel-FwTLR9	TCAACCTCACATGTTATTCTCATGGTGC	Genotyping
154.NeoDel-RvTLR9	TCAAATATGAACTATGTCATGTAGGTGAC	Genotyping

*Cloning means the oligo DNA was used for cloning of sgRNA. IVT means in vitro transcription. For genotyping of Tlr9^−/−^ mice, Tlr9-extra and Tlr9-WT were used for the detection of WT allele; Tlr9-extra and Tlr9-KO-Rv1 were used for the detection of KO allele*.

### Cell Staining

For staining of STAT1 in splenocytes, spleens were obtained from mice and prepared into single cell suspension. RBCs were lysed by RBC lysis buffer (BioLegend) and left cells were stimulated by 2,000 U/ml of interferon-αA (IFN-αA), IFN-β, or 200 U/ml of IFN-γ for 30 min. Stimulated cells were washed twice with PBS and incubated with anti-CD16/32 for blocking Fc receptor. After blocking Fc receptor, cells were fixed by BD Cytofix buffer (BD) for 10 min at 37°C. Fixed cells were washed twice with staining buffer and permeabilized with BD Phoslow Perm Buffer III (BD) for 30 min on ice. The cells were washed twice with staining buffer and incubated with anti-STAT1 and anti-pSTAT1 for 30 min at 4°C. Stained cells were washed twice with staining buffer and suspended in staining buffer for flow cytometry.

For staining of TLR9 in splenocytes, the cells were stained with the following sets of antibodies after blocking Fc receptor. B cells, anti-CD19, and anti-CD3; DCs, anti-CD11c, anti-CD11b, anti-I-A/I-E, anti-CD8α, and anti-CD4; monocytes and neutrophils, anti-CD11b, anti-CD11c, anti-CD49b, and anti-Ly6G. After counter staining, the cells were fixed by a fixation/permeabilization buffer (BD) for 20 min at 4°C. The cells were washed with 1× Perm/Wash buffer (BD) and incubated in 500 ng/ml of anti-TLR9 (J15A7) or isotype control IgG1 in 1× Perm/Wash buffer for 30 min at 4°C. The cells were washed twice with 1× Perm/Wash buffer and suspended in staining buffer for flow cytometry.

For staining of Ba/F3 cells, cells were collected and stained with anti-FLAG in intracellular staining buffer. The cells were washed twice with staining buffer and suspended in staining buffer for flow cytometry.

### Flow Cytometry

Prepared cells were subjected to flow cytometry analysis by LSRFortessa X-20 (BD) or FACS Calibur (BD). For detection of PE, a yellow–green laser was used. Flow cytometry data were analyzed using FlowJo software (FlowJo, Ashland, OR, USA).

### Generation of *Unc93b1*^−/−^ Mice

Targeting vector for *Unc93b1* was constructed as shown in Figure 1D. The vector was linearized by NotI, and transfected into JM8A1.N3 ES cells by electroporation. Recombinant ES cells were selected by Southern blotting and subjected to the injection into fertilized eggs. Obtained N0 *Unc93b1*^DEL/wt^ mice were crossed with CAG-Cre transgenic mice and *Unc93b1*^+/−^ mice were generated. *Unc93b1*^+/−^ mice were intercrossed and *Unc93b1*^−/−^ mice were obtained. The primers for genotyping were shown in Table 1B.

### Stimulation of BM-Derived Cells

Bone marrow-derived macrophages (5 × 10^4^ cells/well) or cDCs (1 × 10^5^ cells/well) were seeded in 96-well plate and stimulated with TLR ligands for 24 h. The culture supernatant was collected and subjected to ELISA to detect cytokines.

### ELISA

TNF-α, IL-6, or IL-12p40 were detected by Mouse TNF-α ELISA Ready-SET-Go!, Mouse IL-6 ELISA Ready-SET-Go!, or Mouse IL-12p40 ELISA Ready-SET-Go!, respectively (Thermo Scientific). IFN-α was detected by VeriKine Mouse Interferon Alpha ELISA Kit (PBL Assay Science).

### Immunoprecipitation

Cells were collected and washed twice with ice cold PBS. Washed cells were lysed by lysis buffer [1% Lubrol, 20 mM Tris/HCl (pH 7.4), 150 mM NaCl, 1 mM CaCl_2_, 1 mM MgCl_2_, 10% glycerol and 1× complete inhibitor] and antibody conjugated-beads were added. The samples were incubated at 4°C for 2 h overnight and washed with washing buffer [0.2% Lubrol, 20 mM Tris/HCl (pH 7.4), 150 mM NaCl, 1 mM CaCl_2_, 1 mM MgCl_2_, 10% glycerol] three times. Washed beads were boiled in 2× SDS sample buffer [4% SDS, 20% glycerol, 0.05% bromphenol blue, 125 mM Tris-HCl (pH 6.8) and 10% 2-ME] at 96°C for 5 min and subjected to SDS-PAGE. Anti-TLR9 beads were made by conjugating anti-TLR9 (clone:NaR9). Anti-FLAG beads were purchased from Sigma.

### Immunoblotting

Prepared protein samples were separated in acrylamide gels by SDS-PAGE and transferred onto PVDF membranes. The membranes were blocked by Block Ace (Dainippon-Sumitomo, Japan) and incubated with antibodies. After incubation with HRP-conjugated antibodies, bands were developed with ECL select (GE Healthcare) and the images were taken by LAS500 (GE Healthcare). Alternatively, bands were developed with BCIP/NBT color development substrate (Promega, Fitchburg, WI, USA). For immunoblotting with anti-TLR9, Can Get Signal Immunoreaction Enhancer Solution (TOYOBO, Osaka, Japan) was used. The intensity of the developed bands was quantified by ImageJ software (NIH, Bethesda, MD, USA) (16).

### Plasmid Construction

Toll-like receptor 9 (TLR9) and mutants was amplified by PCR and cloned into retroviral pMXs vector. The constructs for stem cell transduction were cloned into pMX-puro vector. Targeting vectors for mutant mice were constructed from pEZ-FrtLoxDT vector. sgRNA targeting *Ifnar1* gene was cloned into PX458 vector. Prime STAR DNA polymerase series (TaKaRa Bio, Kusatsu, Japan) were used for PCR. The In-Fusion HD cloning kit (TaKaRa Bio) and Rapid DNA Ligation kit (Roche Applied Science) were used for cloning.

pMX series were kindly provided by Dr Kitamura (The University of Tokyo) (17). pEZ-FrtLoxDT vector was kindly provided by Dr. Klaus Rajewsky (Harvard Medical School).pSpCas9(BB)-2A-GFP (PX458) was a gift from Dr. Feng Zhang (Addgene plasmid #48138) (18).

### Retrovirus Transduction

Plasmids were transfected into Plat-E packaging cells 1 × 10^5^ per well with polyethylenimine (Polysciences, Inc., Warrington, PA, USA) or FuGene6. After 2 days of incubation, supernatants were collected as virus suspensions. Ba/F3 cells were transduced by virus suspensions mixed with DOTAP and centrifuged at 2,000 rpm for 1 h.

For stem cell transduction, mice were treated by 5 mg/head of 5-fluorouracil for 4 days and BM stem cells were harvested from shin bones and thigh bones. The cells were cultured in DMEM containing 15% FBS, 1 mM sodium pyruvate, PS/Gln, 50 µM 2-ME, 100 ng/ml stem cell factor, 10 ng/ml IL-6, and 10 ng/ml IL-3 for 48 h. The medium was discarded and the cells were mixed with retrovirus suspension and DOTAP. The cells were centrifuged at 2,000 rpm for 1 h and cultured for 24 h. The culture medium was changed to fresh retrovirus suspension and the transduction was repeated. The virus suspension was changed to the culture medium for cDC, and 2 µM puromycin was added to the culture medium 48 h later. The cells were cultured for 10–14 days after second transduction and collected for following assays.

### Generation of TLR9 Mutant Mice

Targeting vector for d13 mutation of TLR9 was constructed as shown in Figure 5A. The vector was linearized by SalI and transfected into JM8A1.N3 ES cells by electroporation. Recombinant ES cells were selected by Southern blotting and subjected to the injection into fertilized eggs. Obtained N0 *Tlr9*^d13/wt^ mice were crossed with CAG-Flp transgenic mice and neomycin cassette was deleted. For generating *Tlr9*^−/−^ mice, N0 *Tlr9*^d13/wt^ mice were crossed with CAG-Cre transgenic mice and obtained *Tlr9*^+/−^ mice were intercrossed. The primers for genotyping were shown in Tables 1C,D.

### *In Vivo* Administration of TLR9 Ligand

2 nmol of CpG-B 1668 and 8 µl of DOTAP in 200 µl PBS was intravenously administrated to 6–12 week-old-mice. Peripheral blood was collected from cheek vein at 0, 1, 3, and 6 h after administration. Serum was separated from blood and subjected to ELISA.

### Statistical Analysis

One-way ANOVA or two-way ANOVA with multiple comparisons were performed by comparing the data with GraphPad Prism software (GraphPad Software, La Jolla, CA, USA). If the *p* value was less than 0.05, the difference was judged as significant.

## Results

### Generation of *Ifnar1*^−/−^ and *Unc93b1*^−/−^ Mice

Expression and responses of TLRs are controlled by a variety of cytokines, signaling pathways, and vesicular trafficking molecules (19). In previous studies, type I interferon (IFN) signaling is shown to play a role in controlling basal expression of TLR7 in several types of immune cells (15, 20–22). Although TLR9 mRNA expression is not changed by the lack of type I IFN signaling (21), little is known about the role of type I IFN signaling in TLR9 protein expression. To investigate the role of type I IFN signaling in TLR9 protein expression and its cleavage, we generated *Ifnar1*^−/−^ mice (Figure 1A). STAT1 phosphorylation was observed by the stimulation with IFN-γ but not IFN-α or IFN-β (Figures 1B,C).

Unc93 homolog B1 is required for TLR trafficking, and its deficiency makes endosomal TLRs uncleaved by the restriction in the ER. Little is known, however, about the role of Unc93B1 in TLR9 protein expression. To investigate the role of Unc93B1 in TLR9 protein expression and its cleavage, we generated *Unc93b1*^−/−^ mice (Figure 1D). TNF-α production in response to the ligands for endosomal TLRs, such as TLR3, TLR7, and TLR9 was not seen in *Unc93b1*^−/−^ macrophages (Figure 1E).

These results verified the abolished function of IFNAR1 or Unc93B1 in the *Ifnar1*^−/−^ or the *Unc93b1*^−/−^ mice, respectively.

### Proteolytic Cleavage of TLR9 Is Controlled by Unc93B1 but Not Type I IFN Signaling

**Figure 2:**
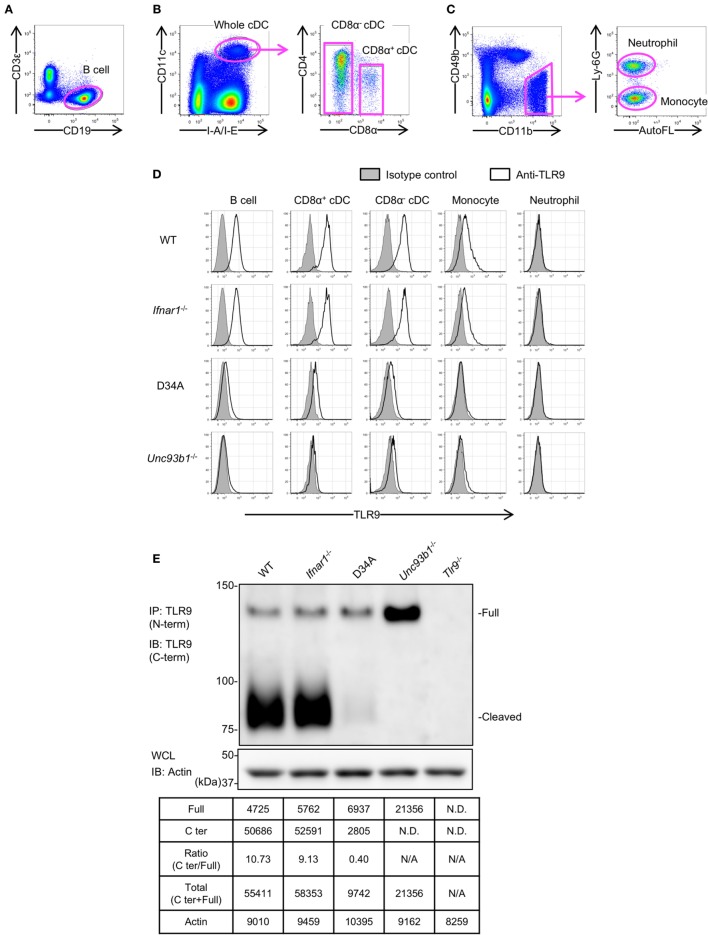
Unc93 homolog B1 (*Unc93b1*) controls the cleavage of toll-like receptor 9 (TLR9) in primary immune cells. **(A–C)** Gating strategy of splenocytes. Splenocytes were stained with indicated antibodies and analyzed by flow cytometry. Gated cells with arrows were drilled down and analyzed by other markers. Whole cDCs were gated out and the other cells were shown in **(C)**. **(D)** Intracellular TLR9 in B cell, CD8α^+^ cDC, CD8α^−^ cDC, monocytes, and neutrophil were stained by anti-TLR9 (J15A7, open histogram) or IgG1 isotype control (tinted histogram). The surface markers of these cells were shown in **(A–C)**. **(E)** Form of full length (Full) or cleaved C-terminal of endogenous TLR9. The endogenous TLR9 of bone marrow-derived cDCs from WT, *Ifnar1*^−/−^, *Unc93b1*^D34A/D34A^, *Unc93b1*^−/−^, or *Tlr9*^−/−^ mice were immunoprecipitated and detected. β-actin in whole cell lysate (WCL) was detected as an internal control. The intensity of the bands was quantified and shown in the table. At least each four mice **(A–D)**, sex, and age matched were used. At least three times of experiments were performed independently **(E)**. Abbreviations: ND, not detected; N/A, not applicable.

We analyzed the expression of endogenous TLR9 protein in the primary immune cells from WT, *Ifnar1*^−/−^, *Unc93b1*^D34A/D34A^, and *Unc93b1*^−/−^ mice by FACS analyses (Figures 2A–D). Neutrophils were analyzed as a negative control (Figure 2D). As a result, the expression of TLR9 was not clearly altered in *Ifnar1*^−/−^ mice (Figure 2D). TLR9 expression in *Unc93b1*^D34A/D34A^ and *Unc93b1*^−/−^ was severely reduced, explaining the deficiency of responses to the TLR9 ligand in these mice (Figures 1E and 2D) (6). A previous report showed that the D34A mutation in Unc93B1 reduces the cleavage of TLR9 (23). To confirm the effect of Unc93B1 deficiency on the cleavage of endogenous TLR9, we performed immunoprecipitation with anti-TLR9 monoclonal antibody. In BM-derived cDCs from *Unc93b1*^D34A/D34A^ mice (D34A mice) and *Unc93b1*^−/−^ mice, TLR9 cleavage was impaired (Figure 2E). Uncleaved TLR9 increased in *Unc93b1*^−/−^ cDCs, but not in *Unc93b1*^D34A/D34A^ cDC, suggesting that uncleaved TLR9 protein is unstable and degraded in *Unc93b1*^D34A/D34A^ cDCs. Even in *Unc93b1*^−/−^ cDCs, it is possible that uncleaved TLR9 is degraded, because FACS analyses showed that TLR9 protein expression is reduced in both *Unc93b1*^−/−^ and *Unc93b1*^D34A/D34A^ cDCs (Figure 2D).

These results suggest that TLR9 protein expression is determined not just by the transcription from mRNA but also by protein degradation.

### Mutation on the Cleavage Sites of TLR9 Reduces the Response to TLR9 Ligand

**Figure 3:**
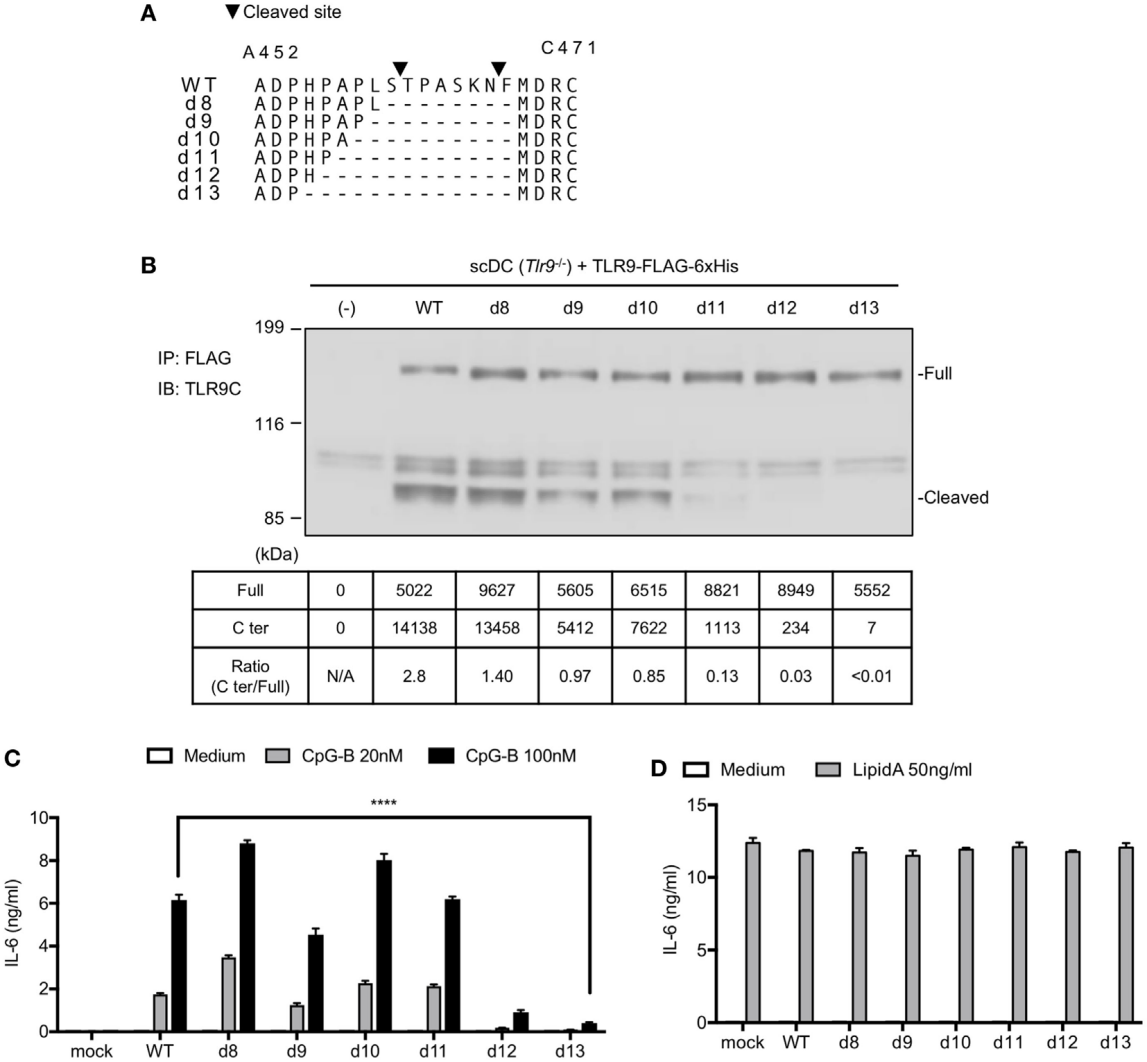
Mutation on the cleavage site of toll-like receptor 9 (TLR9) decreases cleaved TLR9 and its response. **(A)** Amino acid sequence of WT or deletion mutant of TLR9 around cleaved sites. Triangles in the figure indicate the cleaved sites. The cleaved sites were analyzed by Edman degradation. **(B)** Cleavage pattern of TLR9 with mutation in the cleavage site. FLAG-6xHis tagged-WT or mutant TLRs were expressed in stem cell-derived DCs (scDCs) obtained from *Tlr9*^−/−^ mice. FLAG tag was precipitated and C-terminal region of TLR9 was detected. The intensity of the bands was quantified and shown in the table. **(C,D)** scDCs expressing WT or mutant TLR9 were stimulated by TLR9 or TLR4 ligand. After 24 h incubation, IL-6 in culture medium was detected by ELISA. Cells were stimulated in triplicated wells and the mean SD is shown. At least two times of experiments were performed independently **(B–D)**. Data were statistically analyzed by two-way ANOVA with multiple comparisons **(C,D)**. ****p < 0.0001. Abbreviations: N/A, not applicable.

We previously reported that the N-terminal amino acid sequence of cleaved TLR9 fragment starts at T461 or F467 (12). According to the result, we constructed several TLR9 mutants to make TLR9 resistant to cleavage (Figure 3A). These mutants were expressed in *Tlr9*^−/−^ BM stem cell-derived cDCs (scDCs) to ask whether the cleavage of TLR9 is required for TLR9 responses. We studied 8–13 amino acids deletion from F460 to the N-terminal end. The amount of cleaved C-terminal fragment was gradually decreased by increasing the length of deletion (Figure 3B). TLR9-dependent cytokine production was decreased by deleting 12 or 13 amino acids (Figure 3C). Lipid A-dependent cytokine production was not altered by TLR9 mutation (Figure 3D). These results suggest that uncleaved TLR9 fails to respond to its ligand.

**Figure 4:**
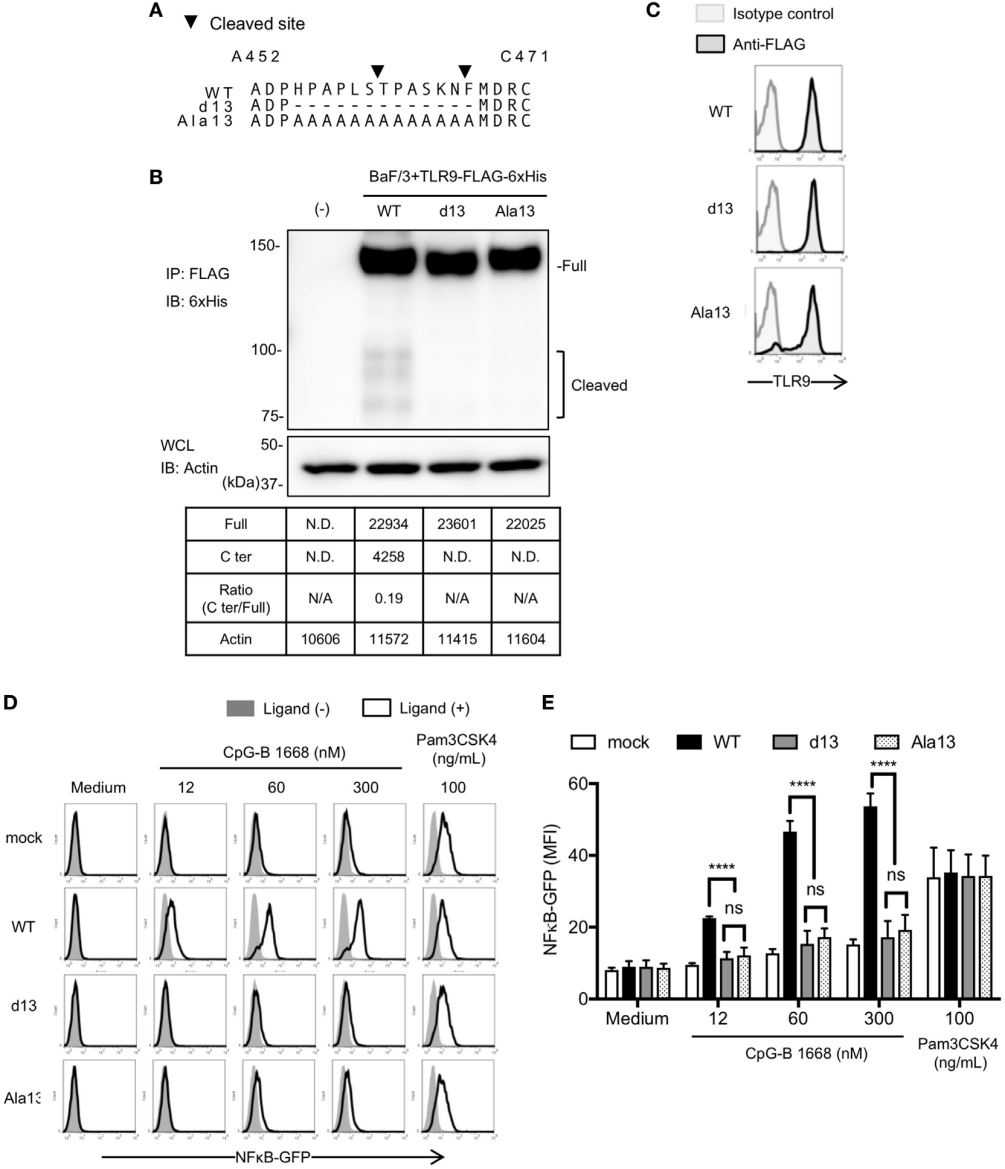
Cleavage of toll-like receptor 9 (TLR9) is dependent on the amino acid sequence around the cleavage sites. **(A)** Amino acid sequence of WT, d13, or Ala13 TLR9. Triangles in the figure indicate the cleaved sites. **(B)** Cleavage pattern of TLR9 with mutation in the cleavage site. FLAG-6xHis tagged-WT or mutant TLR9s were expressed in Ba/F3 cells. FLAG tag was precipitated and C-terminal region of TLR9 was detected by anti-6xHis. β-actin in whole cell lysate (WCL) was detected as an internal control. The intensity of the bands was quantified and shown in the table. **(C–E)** Response of TLR9s in Ba/F3 cells. FLAG-6xHis tagged-WT or mutant TLR9s were expressed in Ba/F3 cells harboring nuclear factor kappa B (NF-κB)-GFP reporter gene. Expression of TLR9s was detected by flow cytometry **(C)**. Cells were stimulated by TLR9 or TLR2 ligand and activation of NF-κB was monitored as GFP expression by flow cytometry after 24 h incubation **(D)**. Mean fluorescent intensity of NF-κB-GFP was indicated as graph. Cells were stimulated in triplicated wells and the mean SD is shown **(E)**. At least three times of experiments were performed independently **(B–E)**. Data were statistically analyzed by two-way ANOVA with multiple comparisons **(D,E)**. ****p < 0.0001. Abbreviations: ns, not significant; ND, not detected; N/A, not applicable.

Next, we also changed these 13 amino acids to alanine (Ala13) to exclude the possibility that the defective TLR9 response is due to altered conformation by deletion of amino acids (Figure 4A). WT TLR9 and its mutants were expressed in pro-B cell line Ba/F3 and their responses were detected by NFκB-GFP reporter assay. As a result, d13 mutant was not cleaved in Ba/F3 cells likewise scDCs (Figure 4B). According to the data, Ala13 was compared to d13 and the cleavage of Ala13 was decreased as much as d13 (Figure 4B), although the expressions of these TLR9s were not different (Figure 4C). TLR9-dependent nuclear factor kappa B (NF-κB) activation were also significantly reduced in d13 and Ala13 mutants (Figures 4D,E). These data suggest that the 13 amino acids around cleaved site are required for the proteolytic cleavage of TLR9 and that uncleaved TLR9 fails to respond to ssDNA.

### Responses to TLR9 Ligand Were Reduced in *Tlr9*^d13/d13^ Mice

To confirm that the d13 mutation on the cleavage site of TLR9 is also important for the response of endogenous TLR9, we generated *Tlr9^d13/d13^* mice. These mice were designed to introduce d13 mutation in the exon2 (Figure 5A). After germline transmission, we confirmed that the *Tlr9* gene lacks 13 amino acids as designed (Figure 5B). By crossbreeding with mice expressing CRE, conventional *Tlr9*^−/−^ mice were also generated (Figure 5A).

Next, we obtained BM-cDCs from WT, *Tlr9^d13/d13^*, or *Tlr9*^−/−^ mice and examined endogenous TLR9 by immunoprecipitation and immunoblotting. Although the cleaved C-terminal region of TLR9 was still observed in *Tlr9^d13/d13^* BM-cDCs, the ratio of C-terminal region of TLR9 over uncleaved TLR9 was drastically reduced from 14.7 in WT mice to 4.3 (Figure 5C). Reflecting the reduction of the cleavage, the response to TLR9 ligand, both of CpG-A and CpG-B was reduced in BM-cDCs from *Tlr9^d13/d13^* (Figure 5D).

**Figure 6:**
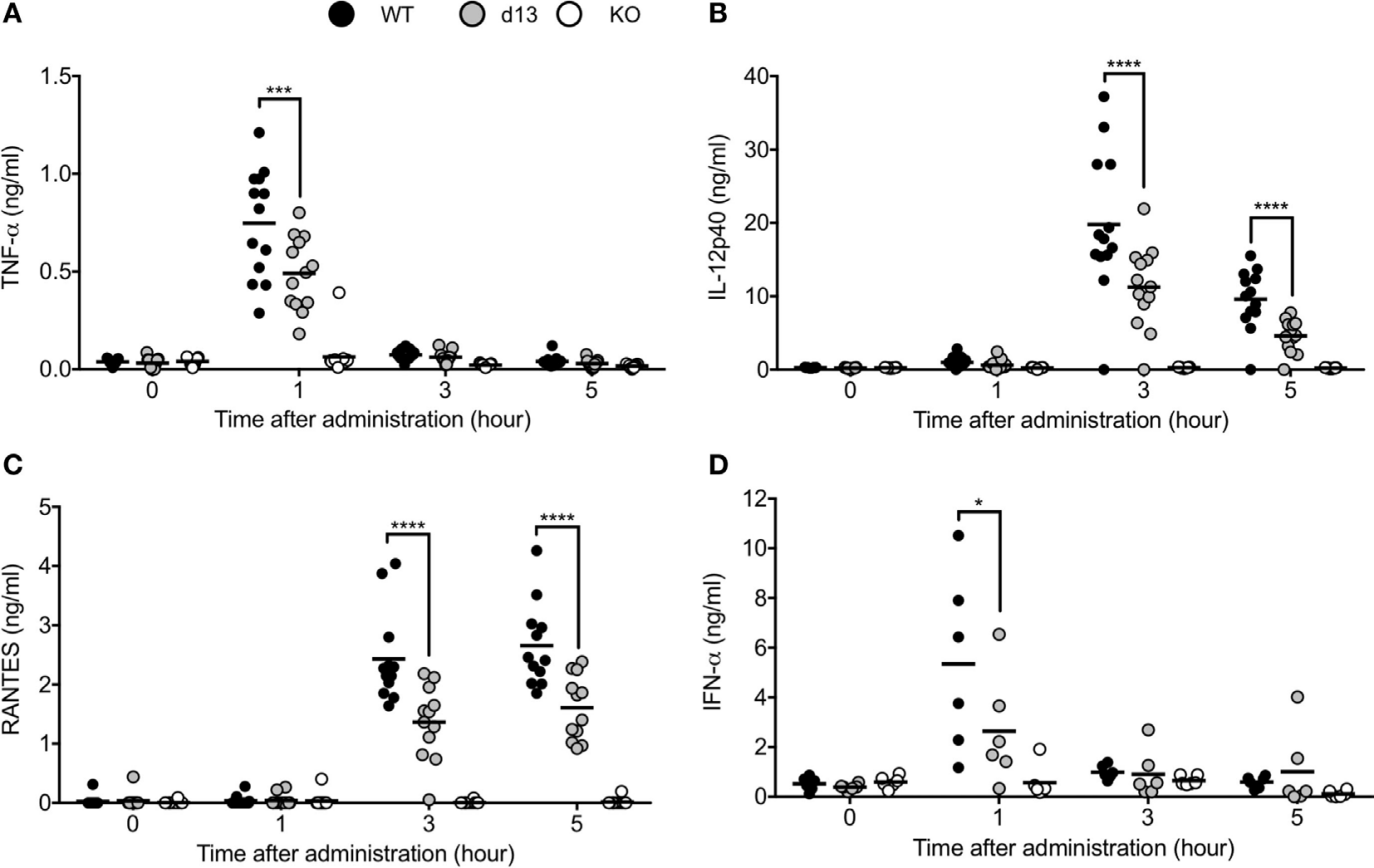
Non-cleaved mutation of toll-like receptor 9 (TLR9) reduces the response of TLR9 *in vivo*. **(A–D)** 2 nmol of CpG-B 1668 and 8 µl of DOTAP were administrated into WT (black circle), *Tlr9*^d13/d13^ (gray circle), or *Tlr9*^−/−^ mice (open circle). Serum was corrected at 1, 3, and 5 h after administration or pre-administration. TNF-α **(A)**, IL-12p40 **(B)**, RANTES **(C)**, and IFN-α **(D)** in the serum were detected by ELISA. Wells of ELISA were triplicated and the mean score of each mouse is shown as a circle. At least 13 **(A–C)** or 6 mice **(D)** were used and analyzed independently. Data were statistically analyzed by one-way ANOVA with multiple comparisons. Bars in the dot graphs indicate the average of score **(A–D)**. ****p < 0.0001, ***p < 0.001, *p < 0.05.

Finally, we injected TLR9 ligand CpG-B into WT, *Tlr9^d13/d13^*, or *Tlr9*^−/−^ mice and measured cytokine/interferon production in serum. TNF-α and IFN-α were detected in serum 1 h after administration, and IL-12p40 or RANTES were detected from 3 h after administration (Figures 6A–D). Production of these cytokines was undetectable in *Tlr9*^−/−^ mice, and significantly impaired in *Tlr9^d13/d13^* mice (Figures 6A–D). Time course of cytokine/interferon production in *Tlr9^d13/d13^* mice was not altered (Figures 6A–D). These results demonstrate that TLR9 proteolytic cleavage is required for TLR9 responses *in vivo*.

## Discussion

In this study, we generated *Tlr9*^d13/d13^ mice harboring the 13 amino acids deletion at the cleavage site of TLR9. In *Tlr9*^d13/d13^ mice, TLR9 cleavage in cDCs was impaired and serum proinflammatory cytokines after TLR9 ligand administration was significantly reduced. These results demonstrate that the proteolytic cleavage of TLR9 ectodomain is required for *in vivo* TLR9 responses. We also generated *Unc93b1*^−/−^ mice and confirmed that cleavage of TLR9 was abolished by Unc93B1 deficiency. As described below, the difference and similarity of the cleavage patterns of TLR9 among these mice and *Unc93b1*^D34A/D34A^ mice help us to study the stability and signaling pathway of endogenous TLR9.

Comparing the data of cleaved TLR9, the cleavage of endogenous TLR9 is more sensitive than overexpressed TLR9. Whereas the ratio of C-terminal against full length of overexpressed WT TLR9 was 0.19 in Ba/F3 cells and 2.8 in scDCs (Figures 3B and 4C), the ratio of cleaved endogenous WT TLR9 in BM-cDCs was more than three times of overexpressed TLR9 (Figures 2E and 5C). Given that the cleavage of overexpressed TLR9 is also different between Ba/F3 cells and scDCs, the effective cleavage of TLR9 varies with type of cells.

Interestingly, the volume of full length TLR9 in d13 BM-cDC is not increased by the reduction of C-terminal fragment (Figure 5C). These results are different from *Unc93b1*^−/−^ cDCs and similar to *Unc93b1*^D34A/D34A^ cDCs (Figure 2E). In *Unc93b1*^−/−^ cDCs, TLR9C is undetectable and the intensity of full length of TLR9 is drastically increased. These results suggest that "pre-cleaved" TLR9 in the ER is protected from degradation and detected as the signal of full length, but "non-cleaved" TLR9 in endolysosome is sensitive to degradation. In other words, full length TLR9 is more sensitive to the degradation in endolysosome than the complex of N-terminal and C-terminal fragments. We used a monoclonal anti-TLR9 "NaR9" bound to N-terminal region of TLR9 for immunoprecipitation (20), thus the C-terminal fragment detected by immunoblotting is the part of the complex of N-terminal and C-terminal fragments of cleaved TLR9. These data imply that the cleaved N-terminal fragment but not N-terminal region in non-cleaved TLR9 stabilizes C-terminal region in endolysosome.

About physiologic relevance with proteolytic cleavage of TLR9, reduction of cleaved TLR9 would enhance the risk of infection due to TLR9 hyporesponsiveness. Among DNA virus, herpes simplex virus (HSV) is recognized by TLR9, which induces type I interferon in pDCs (24–26). Since the induction of IFN-α with CpG-B administration was weak in *Tlr9*^d13/13^ mice, there is a possibility that mutation of the genes for TLR9 cleavage elevates the sensitivity to HSV.

Toll-like receptor 9 (TLR9) is also known as a factor of autoimmune diseases, because TLR9 recognizes self-derived DNA and induces sterile inflammation. For example, psoriasis, type 1 diabetes, and nonalcoholic steatohepatitis are suggested as TLR9-related diseases (27–30). While direct inhibition of TLR9 is a feasible strategy against these diseases, TLR9 cleavage is also expected as a target of TLR9-related diseases. Candidate of the target is cathepsin family, a group of proteases required for the cleavage and response of TLR9 (9, 10, 31). Although the direct effect on TLR9 cleavage is not displayed, cathepsin K inhibitor inhibits TLR9 response and attenuates experimental rheumatoid arthritis in mouse model (32).

The cleavage of TLR9 depends on the binding and intracellular transportation by Unc93B1, so that Unc93B1 is thought as an essential molecule for the cleavage and response of TLR9 (5, 23, 33). Interestingly, TLR9 in non-immune cells, for example, cardiomyocytes and neuron is activated without Unc93B1 (34). TLR9 ligands do not activate NF-κB pathway but AMP-activated kinase (AMPK) signaling is activated in these cells to protect these cells from stress (35). Given that TLR9 ligand activates AMPK signaling pathway in the immune cells by the knockdown of Unc93B1, Unc93B1 might control the signaling pathways between NF-κB and AMPK. Although the mechanisms controlling the expression of Unc93B1 is still unclear, it is important to clarify the relation between TLR9 signaling and metabolism in immune cells. Proteolytic cleavage of TLR9 might be a key to find new mechanisms controlling the multiple pathway of these signaling.

If AMPK signaling is enhanced in *Tlr9*^d13/13^ or *Unc93b1*^−/−^ mice, metabolism might be changed in these mice because AMPK strongly contributes to control metabolism *in vitro* and *in vivo* (36–38). Induction of metabolic syndrome, such as type 2 diabetes or nonalcoholic fatty liver disease, should be examined by using *Tlr9*^d13/13^ or *Unc93b1*^−/−^ mice as further study. Despite the risk of infection by the reduction of TLR9 cleavage, TLR9 or Unc93B1 has a potential to be a target of intervention/prevention against metabolic syndrome.

## Ethics Statement

This study was carried out in accordance with the recommendations of the guidelines of the Animal Research Committee of the Institute of Medical Science, The University of Tokyo (IMSUT). The protocol was approved by the Animal Research Committee of IMSUT (permission number from the committee: A17-82, A17-83, and A17-84).

## Author Contributions

RF, CY, FM, MO, YM, TH, NT, and KM designed and performed experiments. RF, TS, AK, NY, and KM generated mutant mice. CY, RF, and KM wrote manuscript. CY and RF contributed equally.

## Conflict of Interest Statement

The authors declare that the research was conducted in the absence of any commercial or financial relationships that could be construed as a potential conflict of interest.

## Abbreviations

TLR, toll-like receptor; ssDNA, single-stranded DNA; ER, endoplasmic reticulum; Unc93B1, Unc93 homolog B1; NA, nucleic acid; LRR, leucine-rich repeat; IFNAR1, interferon alpha receptor 1; DC, dendritic cell; BM, bone marrow; NF-κB, nuclear factor kappa B; AMPK, AMP-activated kinase; HSV, herpes simplex virus.
